# A do‐it‐yourself approach to waste‐heat recovery in MRI

**DOI:** 10.1002/mrm.70016

**Published:** 2025-07-25

**Authors:** Rüdiger Brühl, Hannes Dillinger, Dirk Trepte, Bernd Ittermann

**Affiliations:** ^1^ Physikalisch‐Technische Bundesanstalt Braunschweig and Berlin Germany

1

Radiology departments contribute substantially to a hospital's carbon footprint,^1^, ^2^, ^3^ and the modality with the worst record is typically MRI.^3^, ^4^, ^5^ The electrical energy consumed by a scanner ends up as waste heat in its internal cooling‐water circuit, and additional money and energy are normally spent to dispose of that waste heat (such as via a secondary house cooling circuit). Attempts to re‐use this heat are rarely made, arguing that the cooling water's temperature level would be too low and the heat flow too volatile for secondary use. Here, we report a do‐it‐yourself approach to waste‐heat recovery,^6^ which was implemented in February 2024, a few months after a new 3T scanner (Cima.X; Siemens Healthineers) was installed at our site.

The power consumption of the scanner in various states of idleness was measured with a power meter (PQA 435; Fluke Corporation) at the circuit breaker panel, yielding 27.5 kW in “scanner ready,” 11.1 kW in “eco,” 7.4 kW in “stand‐by,” and 5.5 kW in “sleep” mode. In eco mode, which is entered automatically, the gradient power amplifiers are turned off, and the system's two internal water pumps are slowed down. In stand‐by mode, which must be entered manually, everything is shut off except the He compressor (5.7 kW) and the water pumps (1.7 kW). In sleep mode, entered automatically, the He compressor cycles between 8‐min on and 4‐min off states, reducing its average power consumption to 3.8 kW. These 3.8 kW were targeted for recovery, as this heat is available 24/7 and comes at a relatively high temperature level of 30ºC or 36°C, depending on the pump speed.

Figure 1A shows a schematic of the original hydraulic components. In the scanner's cooling circuit, the return water from all system components is collected in one common pipe and then pumped through the heat exchanger (HE‐1) with the house cooling circuit. A motor‐controlled bypass valve keeps its outflow temperature at the target value of 24°C. Air flow and temperature in our 140‐m^2^ MRI building are controlled independently by a site‐installed cabinet (ASU‐300‐CW; Stulz) equipped with an electrical heater and a water‐to‐air heat exchanger (HE‐2) for cooling.

**FIGURE 1:**
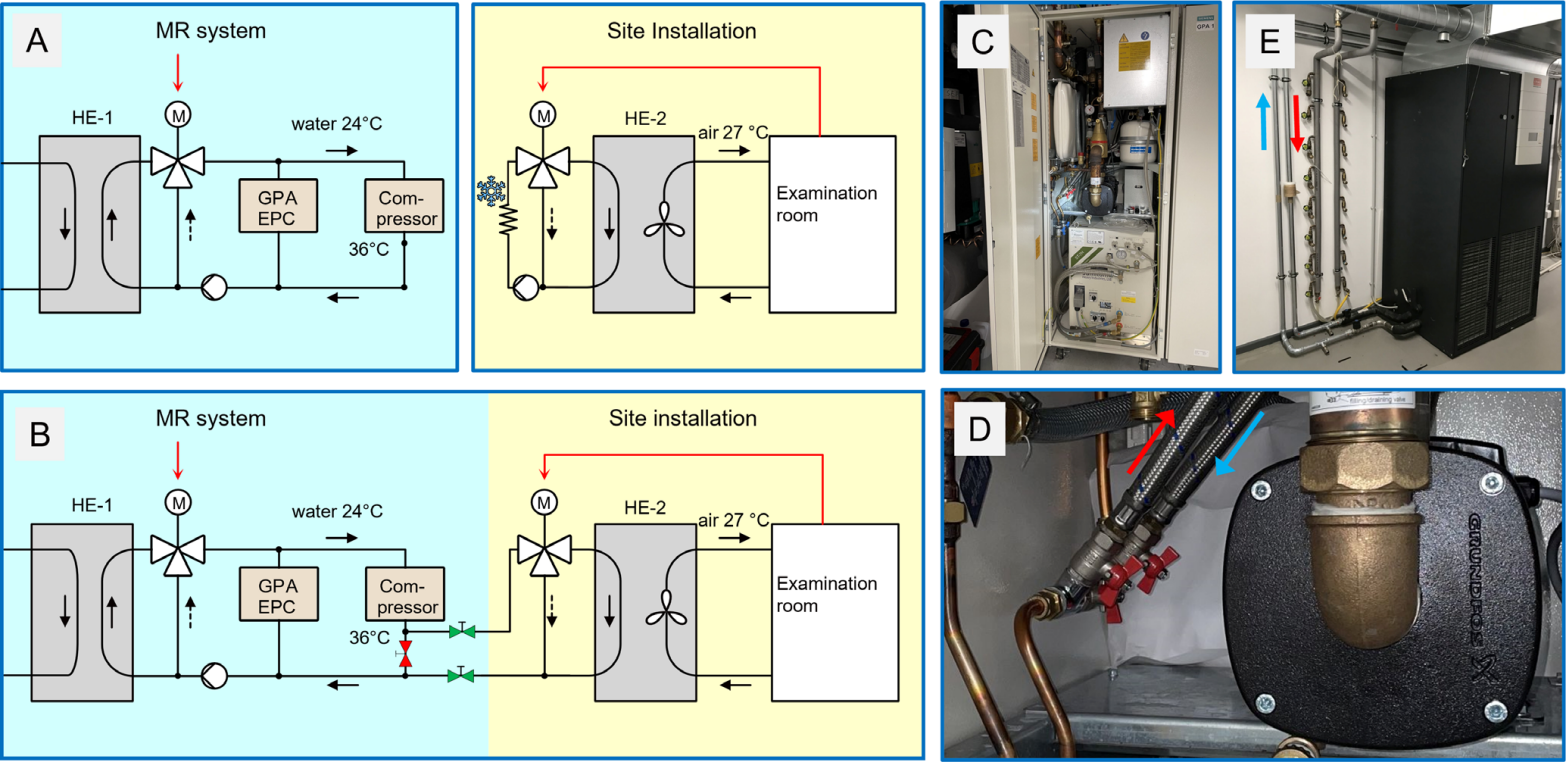
(A) Schematic of the water‐cooling system before modification. (B) After modification, the return water from the compressor is fed through HE‐2 before being pumped back through HE‐1. In normal operation, the red stopcock is always closed, and the two green stopcocks are always open. The cooling water leaves the compressor at 30°C when the scanner is operational and at 36°C when the pump speed is reduced. (C) System cabinet containing compressor, HE‐1, and the system water pump. (D) Close‐up of the three stopcocks and the two flex pipes intercepting the copper pipe from the compressor outlet. (E) Ventilation cabinet with the extension pipes from the He compressor entering at the bottom. EPC, scanner electronics cabinet; GPA, gradient power amplifier; HE‐1, water‐to‐water heat exchanger to the house cooling circuit; HE‐2, water‐to‐air heat exchanger in the ventilation cabinet; M, motor‐controlled bypass valve.

This system was modified as depicted in Figure 1B: After inverting the control mode of the HE‐2 bypass valve, the return water from the He compressor is intercepted and fed to HE‐2 in the ventilation cabinet before it is returned to the scanner cooling circuit. Inside the system cabinet that houses the He compressor (Figure 1C), three stopcocks (Figure 1D) were inserted into the return‐water pipe from the compressor: one to block the direct return path, and two to open the detour loop through the ventilation cabinet (Figure 1E) about 4 m away. Note that the return water from the detour loop is colder than before, so there is no added risk for the scanner. The scanner vendor knew about our modification but was not actively involved in it; they neither supported nor obstructed our work.

The costs for this procedure were about 500 Euro worth of plumbing material plus 3 h of labor. On cold days, temperatures as low as 24°C were measured in the return water from HE‐2 (i.e., 100% of the compressor's waste heat were extracted). Without any operating costs, a total of 33 MWh/a is theoretically available; therefore, while only an estimated 12.5 MWh/a are needed to heat our MRI building all year long, the existing conventional heating was no longer used. With heating costs around 100 €/MWh these days in Germany, financial break‐even was reached somewhere in the middle of the first winter. Additional savings by the reduced heat load to the house cooling circuit exist but cannot be quantified. During 1 year of operation, we never encountered any issues with our setup. Currently, we are preparing an extension to support the floor heating of three adjacent laboratories, which will allow us to recover all the available waste heat.

A waste‐heat recovery system was installed by a simple modification of an MRI scanner's cooling circuit and now provides all the room heating for a small MR site. Water temperatures of 30ºC to 36°C are sufficient for that purpose, and after a few months the accumulated savings already exceeded the installation costs. Because all installations are different, it is unlikely that our solution can be applied identically at other sites. We are confident, however, that with only minor modifications it can be adapted to work in a large variety of environments.

## Data Availability

Data sharing not applicable to this article as no datasets were generated or analysed during the current study.
